# Designing, optimization, and validation of whole blood direct T-ARMS PCR for precise and rapid genotyping of complex vertebral malformation in cattle

**DOI:** 10.1186/s12896-021-00696-5

**Published:** 2021-05-22

**Authors:** R. R. Alyethodi, U. Singh, S. Kumar, R. Alex, G. S. Sengar, T. V. Raja, R. Deb, B. Prakash

**Affiliations:** 1 Animal Science Division, ICAR-Central Island Agricultural Research Institute, Garacharma, Andaman and Nicobar Islands 744101 India; 2 Animal genetics & Breeding Division, ICAR-Central Institute for Research on Cattle, Meerut, UP India; 3 Animal genetics & Breeding Division, ICAR-National Dairy Research Institute, Karnal, Haryana India; 4 ICAR-National Research centre on Pig, Guwahati, Assam India

**Keywords:** T-ARMS PCR, Genetic testing, Direct T-ARMS PCR, Blood, Heparin, EDTA, DNA polymerase, Microcard, SNP analysis, rs438228855

## Abstract

**Background:**

DNA testing in the cattle industry undergoes multiple hurdles. Successful genotyping involves the transportation of samples from the field to the laboratory in a chilled environment followed by DNA extraction, and finally, a specific genotyping protocol is followed. Various researches are focused on overcoming these issues. Microcards offer blood transportation at ambient temperature. Direct PCR methods can save the time of DNA extraction but available only for simplex PCR. Tetra Primer-Amplification Refractory Mutation System based Polymerase Chain Reaction (T-ARMS PCR) can make DNA testing faster in a low-cost setting. The present study was aimed to design, optimize, and validate a T-ARMS PCR for faster DNA testing of SNP responsible for Complex Vertebral Malformation (CVM)-an important genetic disease of the cattle industry. Further, a direct T-ARMS PCR from whole blood was developed to avoid the DNA extraction steps. Lastly, using the optimized protocol, genotyping of blood spotted on Microcard eliminates the need for cold chain maintenance in the transportation of samples.

**Results:**

The present study demonstrated a novel T-ARMS PCR-based genotyping of the SNP rs438228855, which is responsible for CVM. Here, wild genotypes were recognized by 389 bp and 199 bp bands in agarose gel, while the carrier genotype showed an additional 241 bp band. The developed protocol was validated using PCR-Primer Introduced Restriction Analysis (PCR-PIRA) and sequencing. The present study further established a direct T-ARMS PCR for this SNP from whole blood. Different conditions such as heparin and EDTA treated blood, the need for pre-treatment, and two different DNA Polymerases for the direct PCR were optimized. Finally, our optimized protocol successfully genotyped the whole blood samples dried on Insta™DNA cards.

**Conclusions:**

The present study reported the usefulness of primer modified T-ARMS PCR for detecting CVM for the first time. To the best of our knowledge, direct PCR in T-ARMS PCR has never been reported. Lastly, the use of microcards in the developed protocol can make the assay useful in the DNA testing of field samples.

**Supplementary Information:**

The online version contains supplementary material available at 10.1186/s12896-021-00696-5.

## Background

Complex vertebral malformation (CVM) is an important inherited lethal defect of the Holstein population and its crossbreeds worldwide, which results in the production of malformed calves that are either spontaneously aborted or die shortly after birth [1]. Since it is an autosomal recessive gene disorder, the diseased phenotype is only expressed if both alleles are present [2]. However, chances of unrecognized dissemination of such defective genes are possible through carrier animals, which is of great concern in cattle breeding as individual bulls can sire thousands of calves in different countries through artificial insemination [3, 4]. The disease is mainly caused by a missense mutation of the bovine solute carrier family 35 member 3 (SLC35A3) gene. It causes the substitution of Valine to Phenylalanine (V180F) and impairs the function of transporter membrane protein UDP-N-acetyl glucosamine [2]. Fast and precise SNP genotyping is of great use in human and animal disease testing. The responsible SNP (rs438228855) for CVM does not create or abolish the restriction site; hence, genotyping via normal PCR-RFLP does not work. Alternate genotyping methods such as allele-specific PCR (AS-PCR) (International Patent WO 02/40709 A2, 2002), High-Resolution Melting Analysis (HRMA) [5], PCR Single-Stranded Conformation Polymorphism (PCR-SSCP) [6], PCR-PIRA [1], and real-time PCR-based genotyping [7] are reported for genotyping of the SNP. Although these methodologies distinguish CVM mutant allele, they have their drawbacks like the need for two amplification reactions for each sample in AS-PCR, lengthy polyacrylamide gel electrophoresis (PAGE) in PCR-SSCP, restriction digestion followed by PAGE in PCR-PIRA, the requirement of costly equipment and reagents in HRMA and real-time based genotyping. Hence, developing an accurate, rapid, economical assay for genotyping of this SNP is essential.

**Fig. 1:**
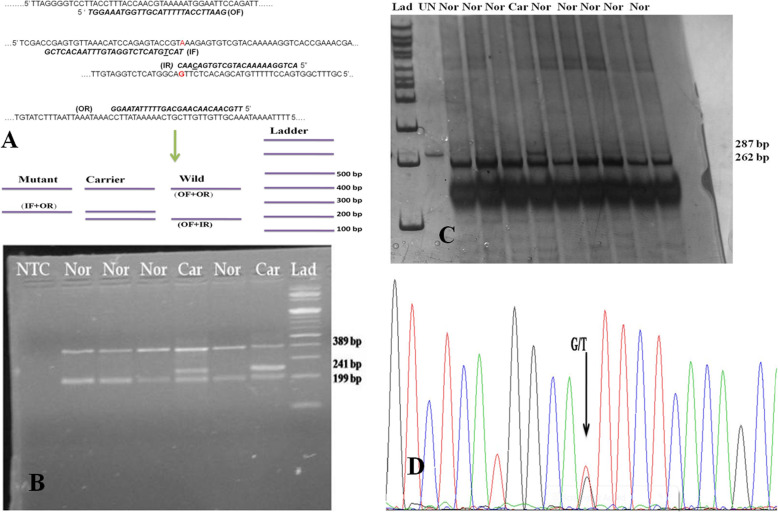
Strategies of development of T-ARMS PCR for Genotyping of SNP rs438228855. **a** Schematic representation of classical T-ARMS PCR, **b** Genotyping of SNP rs438228855 using modified T-ARMS PCR, **c** PCR-PIRA genotyping of SNP rs438228855, **d** Chromatograph file showing heterozygous genotype. Molecular size marker (Lad), Undigested PCR product (UN), Normal genotype (Nor), Carrier genotype (Car), NTC (Non template control)

Tetra Primer-Amplification Refractory Mutation System based Polymerase Chain Reaction (T-ARMS PCR) is a rapid and economical genotyping assay for SNP detection. It involves a single PCR followed by gel electrophoresis [8, 9]. Four primers viz. outer forward (OF), outer reverse (OR), inner forward (IF), and inner reverse (IR) primers, are used in a single tube reaction. The OF/OR primer combination generates the outer fragment of the SNP locus and acts as an internal control for the PCR. The IF/OR and OF/IR primer combinations yield allele-specific amplicons depending on the sample genotype. The inner primers are positioned at a variable distance with the corresponding outer primer so that amplicons of different sizes are generated and distinguished in agarose gel (Fig. 1a) [10]. The use of this method for disease diagnosis in humans [11, 12], including cancer testing [7] and animals [13], has been demonstrated. User-friendly modifications [14] and multiplex variants [7] were attempted for this assay.

The concept of direct PCR, though have wider applications [15, 16], is hampered by the presence of interfering substances such as hemin, bile salts, immunoglobulin G, proteinase, and DNase, and cell lysis detergents [17, 18]. Anticoagulants such as EDTA and heparin interfere with PCR [19]. These inhibitory effects were overcome in different ways by altering the pH of buffer systems [18], optimizing the buffer with Proteinase K [19], altering the buffer chemical composition [20], developing of novel buffer system [17], etc. Genetically engineered polymerase [21], Pfu DNA polymerase, KlenTaq LA, and Real Taq DNA polymerases [22] also helped to remove the inhibition. In simplex PCR, several methods for direct PCR from whole blood exist, while direct amplification for T-ARMS PCR was never attempted. The present study is aimed to develop T-ARMS PCR for genotyping of CVM. Further, the effects of pre-treatment, different anticoagulants, and DNA polymerases were studied and optimized for direct genotyping from the whole blood. The developed protocol was also optimized for genotyping the samples from the blood dried on microcards. These optimizations could enable convenient transportation and faster genotyping of blood samples collected from animals in the field conditions.

## Results

### Development of T-ARMS PCR for genotyping CVM

A T-ARMS PCR was developed for genotyping of rs438228855- SNP responsible for CVM. The outer primers (OF/OR) generated a common 389 bp amplicon. Wild allele-specific primer (IR) in combination with OF and CVM mutant-allele-specific primer (IF) with OR primer produced a 199 bp and 241 bp resolvable amplicons, respectively. Hence, wild genotypes are recognized by the presence of 389 bp and 199 bp bands in agarose gel while the carrier genotype showed an additional 241 bp band (Fig. 1b). Mutant genotype was not detected in the experiment, which otherwise could be identified by the presence of 389 bp and 241 bp bands. The developed protocol was validated for its usefulness using PCR-PIRA. The *NsiI* digestion of PCR–PIRA product (287 bp) yielded fully digested products of 262 and 25 bp in the wild genotypes while an additional undigested 287 band were visible in the carrier genotypes (Fig. 1c). A carrier prevalence of 1.0% was detected in the studied Frieswal population. Sensitivity (the probability that diagnosis is positive among animals which are truly carrier), specificity (the probability that diagnosis is negative among animals which are CVM free), positive predictive value`(PPV) (the probability that the animal is truly carrier if the diagnosis is positive), and negative predictive value (NPV) (probability that the cow is truly non-carrier if the diagnosis is negative) of the test was 100%. This comparison was made against the PCR-PIRA test for CVM, an established methodology for identifying CVM carriers [1]. Further, the Bayesian latent class model [23] iterated using the Markov chain Monte Carlo (MCMC) method to estimate all unknown parameters, including prevalence and accuracy of each diagnostic test, was used in its web-based applications [24]. The developed test’s sensitivity and specificity along with 95% probability intervals were estimated as 92.2 (41.0–100.0) and 99.9 (99.1–100), respectively, whereas the PPV and NPV were 93.0 (45.9–100) and 99.9 (99.0–100) %, respectively. The Bayesian *p* values of the true positive and true negative samples were close to 0.5, indicating the fitness of the Bayesian model on the observed data. Moreover, both the chains in the model converged perfectly, demonstrating the reliability of the estimated parameters. The sequencing results from representative samples further confirmed the variation in identified SNP (Fig. 1d), indicating the developed assay’s precision.

### Development of direct T-ARMS PCR for faster genotyping

#### Effect of pre-treatment

**Fig. 2:**
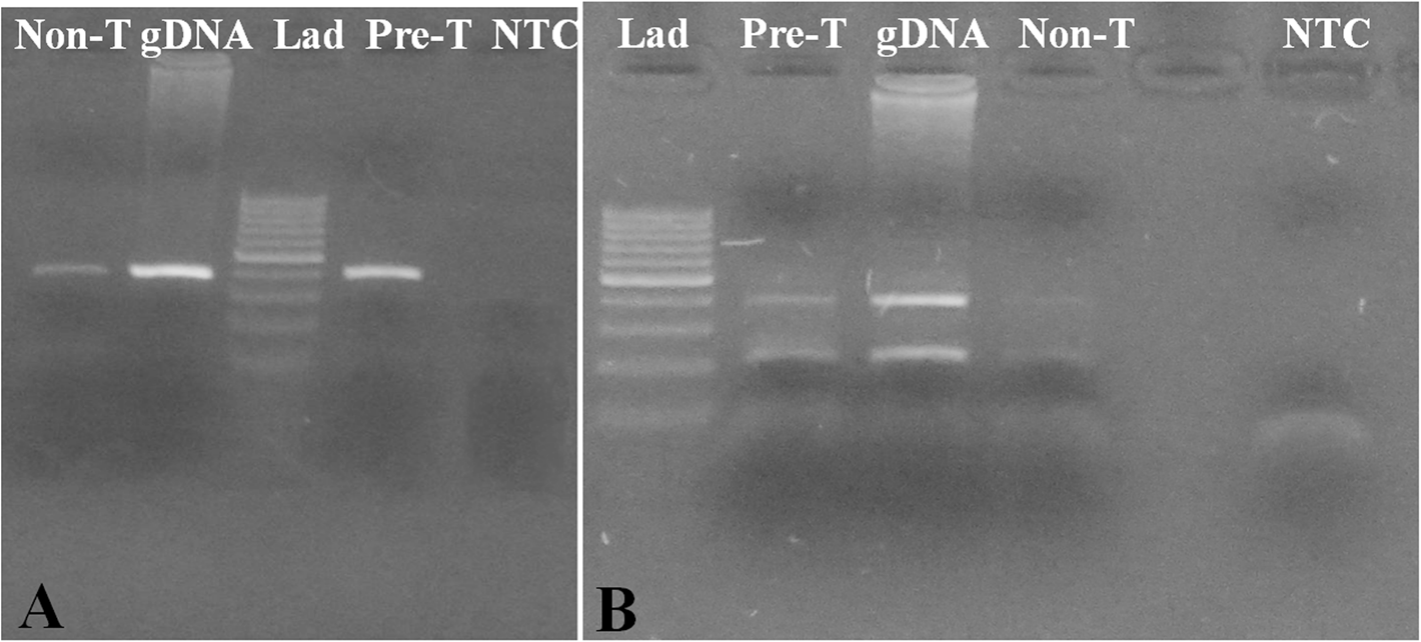
Effect of pre-treatment on Direct simplex PCR and T-ARMS PCR on normal genotype. **a** Direct Simplex PCR **b** Direct T-ARMS PCR. Molecular size marker-100 bp (M), Non-treatment (Non-T), Pre-treatment (Pre-T) genomic DNA (gDNA), NTC (Non template control)

In simplex PCR, amplicons were resolved both in treated and non-treated blood samples (Fig. 2a). The amplicon intensity was lesser in the non-treated sample than in the treated sample. In T-ARMS PCR, only pre-treated blood samples gave detectable amplicons (Fig. 2b).

#### Effect of heparin and EDTA treatment

**Fig. 3:**
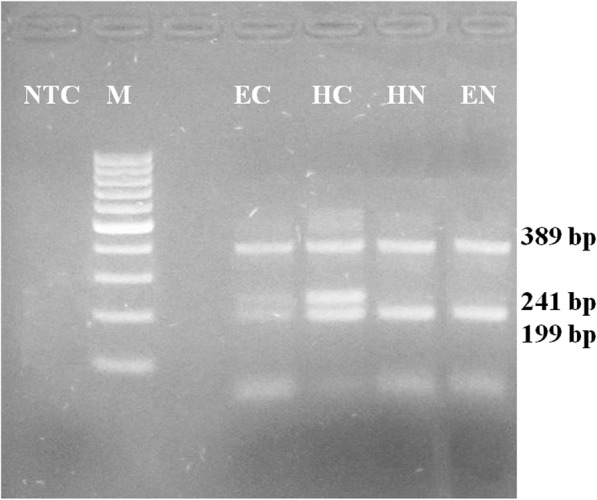
Comparison of Heparin treated and EDTA treated Blood on T-ARMS PCR. Molecular size marker-100 bp (M), T-ARMS Patterns from EDTA treated blood of Carrier (EC), Normal animal (EN), Heparin treated blood of Carrier (HC) and Normal (HN) animals. NTC (Non template control)

Direct T-ARMS PCR from both the heparin-treated and EDTA treated whole blood generated all the expected amplicons as visualized in the gel image (Fig. 3).

#### Influence of reaction mixes on direct PCR

**Fig. 4:**
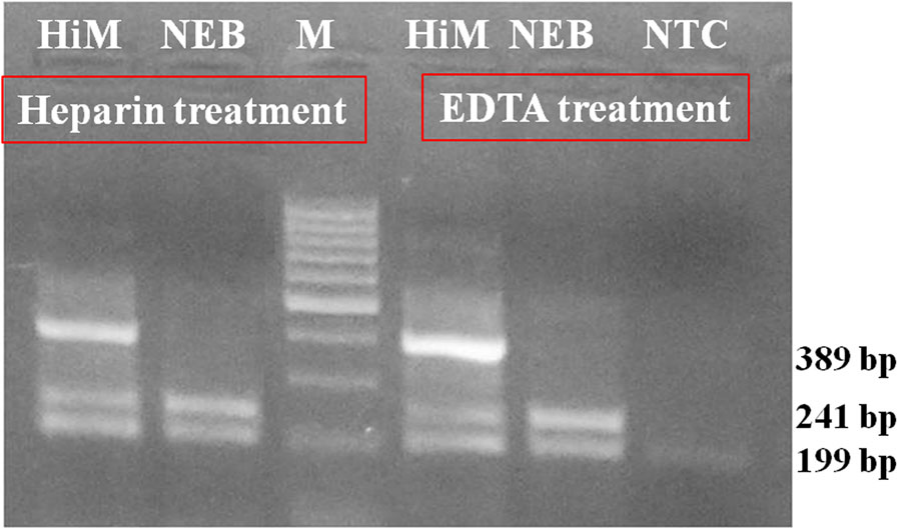
Comparison of three PCR master mixes on Heparin and EDTA treated carrier animal blood sample. Molecular size marker-100 bp (M), Master mixtures of Himedia (HiM), and NEB-Q5 (NEB), NTC (Non template control)

Two different polymerase mixes viz. Taq DNA polymerase (Himedia® - 2X PCR TaqMixture-MBT061) and a high-fidelity polymerase (New England BioLabs® Inc. - Q5® master mix- M0492S) were compared for their beneficial role in direct T-ARMS PCR. Taq DNA polymerase master mix (Himedia®- MBT061) generated all the three expected amplicons from pre-treated whole blood collected in EDTA and heparin (Fig. 4). Q5® master mix (New England BioLabs® Inc- M0492S) generated good intensity resolvable amplicons of inner bands but did not amplify the outer bands (Fig. 4).

#### Direct-T-ARMS PCR and microcards

**Fig. 5:**
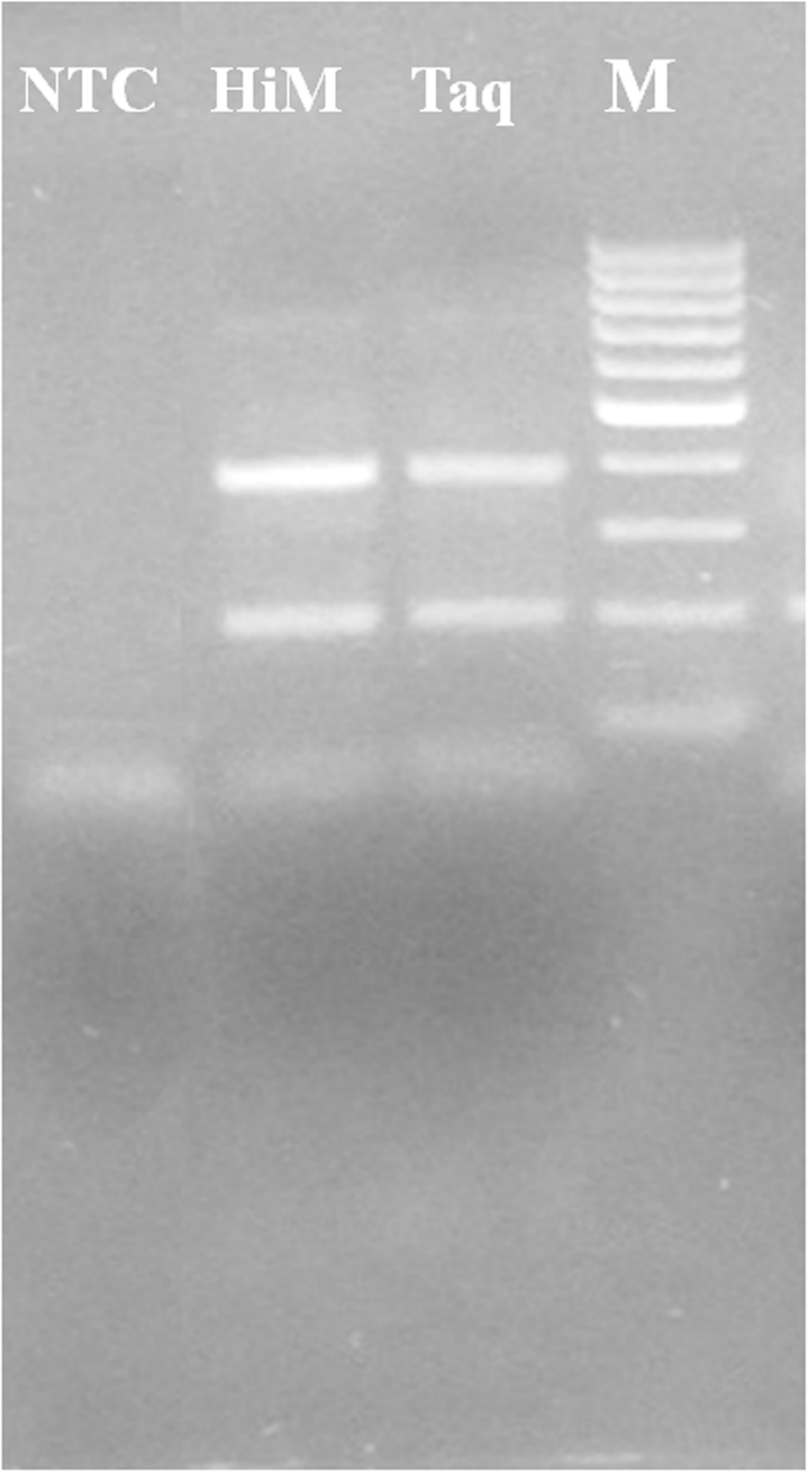
Direct T-ARMS PCR using blood of normal animal dried on Insta™DNA Card. Himedia master mix (Hi) and Sigma Taq polymerase (Sig). Molecular size marker-100 bp (M), NTC (Non template control)

Whole blood dried on Insta™DNA cards showed promising results in Direct T-ARMS PCR (Fig. 5). Both the Taq polymerase from Himedia (MBT061) and Sigma Aldrich (D1806) successfully generated a T-ARMS PCR pattern from blood dried on Insta™DNA cards (Fig. 5).

## Discussion

### T-ARMS PCR for genotyping CVM

The present study reported the usefulness of T-ARMS PCR genotyping to detect CVM for the first time. T-ARMS PCR-based genotyping was published for other important mutations in livestock [25–28], including our report on Bovine Leukocyte Adhesion Deficiency (BLAD) testing [13].

Two rules were modified in the primer designing, which is the most crucial for the successful T-ARMS amplification. Firstly, the position rule where the second primer mismatch of inner primers are always positioned − 2 from the 3′ end of the primer to produce an allele-specific reaction [10]. Secondly, the second primer mismatch is based on the rules that a strong mismatch (G/A or C/T) at the 3′ end of the primer requires a weak mismatch (C/A or G/T) at the position − 2 and vice versa, while a medium-strength mismatch (A/A, C/C, G/G, T/T) requires another medium 2^nd^mismatch [10]. In the present study, the melting temperatures of the primers were given more emphasis. The set criterion was that the melting point of any two primer combinations, which makes the amplicons in PCR (Table.1), should be less than or equal to 2 °C. The present study results demonstrated the possibility of flexing the stringent rules followed by Ye et al., [10] in deciding the second mismatch. Similar attempts of user-friendly modifications to primers were reported [14]. It is reported that the melting temperature is the major deciding factor in the discrimination of alleles in T-ARMS PCR [8, 13]. Different authors’ different criteria showed successful T-ARMS PCR, including this report, which indicated that the primer interactions are complex in T-ARMS PCR. In accordance, it is reported that the interaction between the internal mismatch and nearby nucleotides are influenced by the salts, ions, PCR enhancers, etc., of the reaction mix [29]. These flexing strategies could help opt for suitable strategies to access this methodology in mutations that do not yield a T-ARMS pattern [10] and in the case of GC-rich regions [8].

**Table 1:** Primer sequence and parameters, the nucleotides of inner primers differ between Ye et al., [10] and manually modified are shown in bold, underlined, and italic letters

SNP	Primer	Primer Sequence	Intended Allele	Product size (bp)	Melting temperature (Tm) (°C)	Tm Difference (°C)	Designed
rs438228855 (CVM)	OF	5′ TGGAAATGGTTGCATTTTTACCTTAAG	Both allele	389	54.7	0.5(OF/OR)	Ye et al., 2001 [10]
	OR	5′ TTGCAACAACAAGCAGTTTTTATAAGG			55.2		
	IF	GGCTCACAATTTGTAGGTCTCATGG***T***AT	Mutant	242	58.5	3.3(IF/OR)	
	IR	ACTGGAAAAACATGCTGTGAG***G***AC	Wild	199	57.7	3.0(OF/IR)	
	IF	5′ GCTCACAATTTGTAGGTCTCATG***T***CAT	Mutant	242	57.2	2.0(IF/OR)	Manual modification
	IR	5′ ACTGGAAAAACATGCTGTGA***C***AAC	Wild	199	56.4	1.7(OF/IR)	

### Factors influencing direct T-ARMS PCR

The results of the present study indicate the importance of pre-treatment in T-ARMS PCR. Direct simplex PCR amplification without pre-treatment of whole blood was reported in PCR buffer with higher MgCl_2_ [20], while others reported the need for pre-treatment in direct simplex PCR [30, 31]. The present study demonstrated that, in direct simplex PCR, pre-treatment might not be necessary if a higher MgCl_2_, i.e., 3.5 mM MgCl_2_ is used, and is more beneficial for the intensity of the band is concerned. In the case of direct T-ARMS PCR, pre-treatment is beneficial and necessary.

In the present study, we demonstrated the direct T-ARMS PCR from heparin, and EDTA treated blood samples. Heparin and EDTA have commonly used anticoagulants in blood collection. For molecular biology, EDTA collected samples are processed while the heparin samples are not used since they co-precipitate along with the DNA. If not appropriately processed, heparin, and EDTA inhibit PCR reaction via different mechanisms [32]. The successful demonstration of direct simplex PCR from heparin and EDTA treated blood was reported [18].

The present study indicated that the polymerase enzyme and their buffer system used in PCR affects the direct T-ARMS PCR. This is in concordance with the report that different polymerases and buffer system may behave differently in direct PCR [33]. Compared to commonly used Taq DNA polymerase, Q5 DNA polymerase is a novel polymerase with 3′ → 5′ exonuclease activity and is fused to the processivity-enhancing Sso7d DNA-binding domain to give high fidelity. This may indicate the possibility of inter-lab and intra-lab variation when different Taq-polymerases or buffers are used, which necessitate a prior lab and reagent optimization before performing this assay.

### Direct-T-ARMS PCR using microcards

The present study genotyped blood stains dried on microcards using the complex T-ARMS PCR. Microcards are available from different firms. Its use in collecting, stabilizing, processing, transport, and archiving blood samples is documented [34]. PCR and multiplex PCR using DNA dried on such card or direct card punches are reported [34, 35]. The blood dried on microcards can be shipped at ambient temperature and stored at ambient temperature for more than a year [34, 36]. The minimal storage space and maintenance cost requirement of these cards make them attractive, especially in field sample collection [36]. The blood stain dried on such cards can either directly used for the PCR or processed for DNA extraction. Its use as a laboratory and field sampling device is established [37, 38].

The developed T-ARMS PCR from the blood stains dried on microcards could benefit the cattle industry on multiple grounds viz. blood collection and transportation at ambient temperature using the micro card, and direct T-ARMS PCR genotyping, which removes the need for DNA extraction. Hence the developed protocol used in combination with the micro card makes the disease screening or other SNP screening precise, rapid, and economical. T-ARMS PCR is cost-effective compared to other contemporary protocols such that T-ARMS is a single tube assay where no costly equipment and reagents are involved. Also, the post-PCR manipulations such as PAGE in PCR-PIRA are not needed here. Hence, the time spent is the minimum [8, 39]. This developed methodology can be extended to other SNP genotyping, especially if PCR-RFLP wouldn’t be a genotyping choice.

## Conclusion

The present study reported developing a novel T-ARMS PCR to screen CVM genotypes as a precise, cheaper, and faster alternative to other existing protocols. Further direct T- ARMS PCR from whole blood samples was optimized under different conditions. It can simplify the genotyping process and achieve genotyping with less cross-contamination. The optimized direct PCR on microcards can further speed up the genotyping process and be useful in genotyping field samples.

## Methods

### Development of T-ARMS PCR for genotyping CVM

#### Sample collection and DNA extraction

Two hundred blood samples were collected from Frieswal (HF x Sahiwal cross) bulls reared at the ICAR-Central Institute for Research on Cattle, Meerut, India. Genomic DNA was isolated from the aseptically collected whole blood by the conventional phenol-chloroform method [40] with minor modifications. The study was approved by the Institute Animal Ethics Committee (IAEC) under CPCSEA guidelines. The quality of isolated DNA was assessed by an agarose gel electrophoresis (0.7%). The quantity and purity were measured using NanoDrop spectrophotometer (Thermo scientific). DNA had OD_260/280_, and OD_260/230_ ratio of 1.7–1.9 and 2.0–2.2, respectively, and no smearing detected on agarose gel was used for downstream PCR. The DNA was dissolved in TE buffer (pH 8.0) and kept at − 20 °C until use.

#### Primer designing and SNP analysis

Initially, the T-ARMS primers are designed using T-ARMS software (http://cedar.genetics.soton.ac.uk) [10] on ensemble ENSBTAG00000012454 sequence as per the default parameters of the software. The inner primers were positioned asymmetrically for the common (outer) primers to generate allele-specific amplicons with different product lengths, as shown (Fig. 1a). All generated primers (Table 1) are assessed for their thermodynamic properties using Oligoanalyzer 3.1 software (https://eu.idtdna.com/calc/analyzer). Considering the importance of melting temperature (Tm) on the sensitivity of T-ARMS PCR [8], the inner primers were modified to make the Tm close to its counter primer (forward-reverse). The criterion set was that the Tm difference should be less than or equal to 2 °C between primer combinations that make PCR amplicons in reaction, i.e., OF/OR, IF/OR, IR/OF (Table.1). The primer specificity was tested using the BLAST program of NCBI.

#### In vitro amplification of SNP rs438228855

The T-ARMS PCR reaction was carried out in a final reaction volume of 25 μl. The cocktail comprised of 80 to 100 ng of genomic DNA, 200 μM of each dNTP (Sigma Aldrich-India, DNTP100), 5 picomoles of outer primers (IDT, India) and 10 picomoles of inner primers (IDT India), 1 unit of Taq DNA polymerase (Sigma Aldrich- India, D1806) in buffer containing 10 mM Tris-HCl, pH 8.3, 50 mM KCl, 1.5 mM MgCl_2._ This was further supplemented with 0.25 μl of 50 mM MgCl_2_ to make the final concentration to 2 mM in the reaction mix. DNA was amplified with an initial denaturation at 94 °C for 5 min, followed by 35 cycles comprising of denaturation at 94 °C for 30 s, annealing at 62 °C for 45 s, extension at 68 °C for 35 s, and a final extension 72 °C for 10 min. The generated amplicons were separated in 1.5% agarose gel and visualized under UV light by the AlphaImager gel documentation system. The carrier and normal allele were differentiated by checking the amplicon sizes in reference to 100 bp size markers.

### Validation of the assay

The developed T-ARMS PCR was validated by our earlier published method of PCR-PIRA [4] using the *NsiI* enzyme (NEB India, R0127S). For further comparison, 100 samples which were earlier tested for CVM by PCR-PIRA [22] were added and screened for the presence of carriers using the developed test tetra arms PCR test. Prevalence, sensitivities, specificities, and PPV and NPV estimated by using the conventional method (assuming that PCR-PIRA test is perfect) and imperfect gold standard model (Bayesian latent class model) were estimated using a web-based application [24]. Further, the amplified products using outer forward and reverse primers from the representative samples (carrier and normal) were sequenced with ABI 3100 (Applied Biosystems, USA) automated DNA Sequencer, and the sequences were analyzed for the SNP using the NCBI BLAST program.

### Development of direct T-ARMS PCR for faster genotyping

#### Effect of pre-treatment

Blood samples were pre-treated as described [30] with minor modifications. In brief, 2 μl blood with no anticoagulant, in 1 X Taq buffer, were pre-heated for 95 °C for 5 min followed by 16 alternating heat-cool cycles of 95 °C for 1 min and 50 °C for 1 min. Samples without pre-treatment were used as control. Both simplex and T-ARMS PCR were set in 50 μl volume. The final simplex PCR mix consisted of 2 U of Taq DNA polymerase (Sigma Aldrich, India- D1806), outer primers (0.2 μM), and 3.5 mM MgCl_2_ in IX Taq buffer. Additionally, for T-ARMS PCR, two inner primers (0.4 μM) were added. Genomic DNA (200 ng) was used as a positive template control. The PCR reaction was set up as described.

#### Use of heparin and EDTA treated blood in direct T-ARMS PCR

Whole blood samples were collected aseptically in heparin (Lithium Heparin- BD Diagnostics) and EDTA vacutainer tubes (K3E/EDTA- BD Diagnostics, India). 2 μl heparin treated/EDTA treated blood was added into 23 μl 1X Taq buffer (D1806) and pre-treated as above. Direct T-ARMS PCR was carried out after pre-treatment, as described earlier.

#### Use of different polymerases in direct T-ARMS PCR

Two different polymerases viz. Taq DNA polymerase (2X PCR TaqMixture from Himedia®- MBT061, India), and Q5 high fidelity polymerase (Q5® High-Fidelity 2X Master Mix- from New England BioLabs® Inc- M0492S, India) were used. Blood samples, collected in heparin and EDTA, were added into 23 μl nuclease-free water and pre-treated as described. Enzyme master mixes were added and advanced for the T-ARMS PCR using the reaction set up as described.

### Use of micro card

Blood was placed directly on the Microcard (Insta™DNA cards- Himedia, India) with the precaution of no two drops falls on the same spot as per the manufacturer’s recommendations. Taq DNA polymerases which yielded the best results during the optimization viz. MBT061 and D1806 were used. A two mm sized dried blood punch was cut out and placed in 23 μl nuclease-free water (MBT061) or 23 μl 1X Taq buffer (D1806), pre-treated and advanced for T-ARMS PCR as described.

## Supplementary Information

**Additional file 1.**

## Data Availability

Sequence data used in this study is deposited in the GeneBank under the accession Number MW759068.

## Abbreviations

T-ARMS PCR: Tetra Primer-Amplification Refractory Mutation System PCR
BLAD: Bovine Leukocyte Adhesion Deficienc
AS-PCR: Allele Specific-PCR
HRMA: High-Resolution Melting Analysis
PIRA-PCR: Primer Induced Restriction Analysis-PCR
OF: Outer Forward
OR: Outer Reverse
IF: Inner Forward
IR: Inner Reverse
MgCl_2_: Magnesium Chloride
CVM: Complex vertebral malformation
SNP: Single Nucleotide Polymorphism

## References

[1] Kanae Y, Endoh D, Nagahata H, Hayashi M (2005). A method for detecting complex vertebral malformation in Holstein calves using polymerase chain reaction--primer introduced restriction analysis. J Vet Diagnostic Investig.

[2] Thomsen B, Horn P, Panitz F, Bendixen E, Petersen AH, Holm L-E, Nielsen VH, Agerholm JS, Arnbjerg J, Bendixen C (2006). A missense mutation in the bovine SLC35A3 gene, encoding a UDP-N-acetylglucosamine transporter, causes complex vertebral malformation. Genome Res.

[3] Agerholm JS, Bendixen C, Andersen O, Arnbjerg J (2001). Complex vertebral malformation in Holstein calves. J Vet Diagnostic Investig.

[4] Alyethodi RR, Kumar S, Deb R, Alex R, Singh U, Sharma S, et al. Using PCR-PIRA based genotyping for identifying complex vertebral malformation allele in Frieswal young bulls in India. Iran J Vet Res. 2018;19:44-7. PMC5960772 29805462

[5] Gabor M, Miluchová M, Trakovická A, Riecká Z, Candrák J (2012). Vavriš\’\inová K. detection of complex vertebral malformation carriers in Slovak Holstein cattle by high resolution melting analysis. Acta Vet Brno.

[6] Ruść A, Kamiński S (2007). Prevalence of complex vertebral malformation carriers among polish Holstein-Friesian bulls. J Appl Genet.

[7] Zhang C, Liu Y, Ring BZ, Nie K, Yang M, Wang M, et al. A novel multiplex tetra-primer ARMS-PCR for the simultaneous genotyping of six single nucleotide polymorphisms associated with female cancers. PLoS One. 2013;8:e62126. 10.1371/journal.pone.0062126. 10.1371/journal.pone.0062126 PMC3629144 23614025

[8] Medrano RFV, de Oliveira CA (2014). Guidelines for the tetra-primer ARMS--PCR technique development. Mol Biotechnol.

[9] Zhang S, Dang Y, Zhang Q, Qin Q, Lei C, Chen H, Lan X (2015). Tetra-primer amplification refractory mutation system PCR (T-ARMS-PCR) rapidly identified a critical missense mutation (P236T) of bovine ACADVL gene affecting growth traits. Gene..

[10] Ye S, Dhillon S, Ke X, Collins AR, Day INM (2001). An efficient procedure for genotyping single nucleotide polymorphisms. Nucleic Acids Res.

[11] Mohtavinejad N, Nakhaee A, Harati H, Poodineh J, Afzali M (2015). SIRT1 gene is associated with cardiovascular disease in the Iranian population. Egypt J Med Hum Genet.

[12] Randhawa R, Duseja A, Changotra H (2017). A novel tetra-primer ARMS-PCR based assay for genotyping SNP rs12303764 (G/T) of human Unc-51 like kinase 1 gene. Mol Biol Rep.

[13] Alyethodi RR, Singh U, Kumar S, Deb R, Alex R, Sharma S, Sengar GS, Prakash B (2016). Development of a fast and economical genotyping protocol for bovine leukocyte adhesion deficiency (BLAD) in cattle. Springerplus..

[14] Mesrian Tanha H, Mojtabavi Naeini M, Rahgozar S, Rasa SMM, Vallian S (2015). Modified tetra-primer ARMS PCR as a single-nucleotide polymorphism genotyping tool. Genet Test Mol Biomarkers.

[15] Nishimura N, Nakayama T, Tonoike H, Kojima K, Shirasaki Y, Kondoh K, Yamada T (2002). Various applications of direct PCR using blood samples. Clin Lab.

[16] Ben-Amar A, Oueslati S, Mliki A (2017). Universal direct PCR amplification system: a time-and cost-effective tool for high-throughput applications. 3. Biotech..

[17] Yang YG, Kim JY, Song Y-H, Kim D-S (2007). A novel buffer system, AnyDirect, can improve polymerase chain reaction from whole blood without DNA isolation. Clin Chim Acta.

[18] Bu Y, Huang H, Zhou G (2008). Direct polymerase chain reaction (PCR) from human whole blood and filter-paper-dried blood by using a PCR buffer with a higher pH. Anal Biochem.

[19] Li H, Xu H, Zhao C, Sulaiman Y, Wu C (2011). A PCR amplification method without DNA extraction. Electrophoresis..

[20] Sharma R, Virdi AS, Singh P (2012). A novel method for whole blood PCR without pretreatment. Gene..

[21] Kermekchiev MB, Kirilova LI, Vail EE, Barnes WM (2009). Mutants of Taq DNA polymerase resistant to PCR inhibitors allow DNA amplification from whole blood and crude soil samples. Nucleic Acids Res.

[22] Matheson CD, Gurney C, Esau N, Lehto R. Assessing PCR inhibition from humic substances. Open Enzym Inhib J. 2010;3:38-45.

[23] Hui SL, Walter SD. Estimating the error rates of diagnostic tests. Biometrics. 1980;36(1):167–71. 7370371

[24] Lim C, Wannapinij P, White L, Day NPJ, Cooper BS, Peacock SJ, Limmathurotsakul D (2013). Using a web-based application to define the accuracy of diagnostic tests when the gold standard is imperfect. PLoS One.

[25] Fonseca PAS, Rosse IC, DeMiranda M, Machado MA, Verneque RS, Peixoto M (2013). A new tetra-primer ARMS--PCR for genotyping bovine kappa-casein polymorphisms. Genet Mol Res.

[26] Ahlawat S, Sharma R, Maitra A, Roy M, Tantia MS (2014). Designing, optimization and validation of tetra-primer ARMS PCR protocol for genotyping mutations in caprine Fec genes. Meta Gene.

[27] Singh R, Deb R, Singh U, Alex R, Kumar S, Chakraborti S, Sharma S, Sengar G, Singh R (2014). Development of a tetra-primer ARMS PCR-based assay for detection of a novel single-nucleotide polymorphism in the 5′ untranslated region of the bovine ITGB6 receptor gene associated with foot-and-mouth disease susceptibility in cattle. Arch Virol.

[28] Li M, Sun X, Jiang J, Sun Y, Lan X, Lei C, Zhang C, Chen H (2014). Tetra-primer ARMS-PCR is an efficient SNP genotyping method: an example from SIRT2. Anal Methods.

[29] SantaLucia J, Hicks D (2004). The thermodynamics of DNA structural motifs. Annu Rev Biophys Biomol Struct.

[30] Burckhardt J (1994). Amplification of DNA from whole blood. Genome Res.

[31] McCusker J, Dawson MT, Noone D, Gannon F, Smith T (1992). Improved method for direct PCR amplification from whole blood. Nucleic Acids Res.

[32] Wang J-T, Wang TH, Sheu J-C, Lin SM, Lin JT, Chen D-S (1992). Effects of anticoagulants and storage of blood samples on efficacy of the polymerase chain reaction assay for hepatitis C virus. J Clin Microbiol.

[33] Al-Soud WA, Jönsson LJ, Rådström P (2000). Identification and characterization of immunoglobulin G in blood as a major inhibitor of diagnostic PCR. J Clin Microbiol.

[34] Kline MC, Duewer DL, Redman JW, Butler JM, Boyer DA (2002). Polymerase chain reaction amplification of DNA from aged blood stains: quantitative evaluation of the “suitability for purpose” of four filter papers as archival media. Anal Chem.

[35] Makowski GS, Nadeau FL, Hopfer SM (2003). Single tube multiplex PCR detection of 27 cystic fibrosis mutations and 4 polymorphisms using neonatal blood samples collected on Guthrie cards. Ann Clin Lab Sci.

[36] da Cunha Santos G (2018). FTA cards for preservation of nucleic acids for molecular assays: a review on the use of cytologic/tissue samples. Arch Pathol Lab Med.

[37] Muthukrishnan M, Singanallur NB, Ralla K, Villuppanoor SA (2008). Evaluation of FTA®cards as a laboratory and field sampling device for the detection of foot-and-mouth disease virus and serotyping by RT-PCR and real-time RT-PCR. J Virol Methods.

[38] Sharma S, Kolte SW, Gawande PJ, Kurkure NV, Jadhao SG, Panchbhai CG (2019). A comparative study between blood smear, whole blood PCR and FTA card PCR for diagnosis of Theileria annulata and Theileria orientalis in cattle. J Anim Res.

[39] Alyethodi RR, Singh U, Kumar S, Alex R, Deb R, Sengar GS, Raja TV, Prakash B (2018). T-ARMS PCR genotyping of SNP rs445709131 using thermostable strand displacement polymerase. BMC Res Notes.

[40] Green MR, Hughes H, Sambrook J, MacCallum P (2012). Molecular cloning: a laboratory manual. Molecular cloning: a laboratory manual.

